# Development and validation of the MMCD score to predict kidney replacement therapy in COVID-19 patients

**DOI:** 10.1186/s12916-022-02503-0

**Published:** 2022-09-02

**Authors:** Flávio de Azevedo Figueiredo, Lucas Emanuel Ferreira Ramos, Rafael Tavares Silva, Daniela Ponce, Rafael Lima Rodrigues de Carvalho, Alexandre Vargas Schwarzbold, Amanda de Oliveira Maurílio, Ana Luiza Bahia Alves Scotton, Andresa Fontoura Garbini, Bárbara Lopes Farace, Bárbara Machado Garcia, Carla Thais Cândida Alves da Silva, Christiane Corrêa Rodrigues Cimini, Cíntia Alcantara de Carvalho, Cristiane dos Santos Dias, Daniel Vitório Silveira, Euler Roberto Fernandes Manenti, Evelin Paola de Almeida Cenci, Fernando Anschau, Fernando Graça Aranha, Filipe Carrilho de Aguiar, Frederico Bartolazzi, Giovanna Grunewald Vietta, Guilherme Fagundes Nascimento, Helena Carolina Noal, Helena Duani, Heloisa Reniers Vianna, Henrique Cerqueira Guimarães, Joice Coutinho de Alvarenga, José Miguel Chatkin, Júlia Drumond Parreiras de Morais, Juliana Machado-Rugolo, Karen Brasil Ruschel, Karina Paula Medeiros Prado Martins, Luanna Silva Monteiro Menezes, Luciana Siuves Ferreira Couto, Luís César de Castro, Luiz Antônio Nasi, Máderson Alvares de Souza Cabral, Maiara Anschau Floriani, Maíra Dias Souza, Maira Viana Rego Souza-Silva, Marcelo Carneiro, Mariana Frizzo de Godoy, Maria Aparecida Camargos Bicalho, Maria Clara Pontello Barbosa Lima, Márlon Juliano Romero Aliberti, Matheus Carvalho Alves Nogueira, Matheus Fernandes Lopes Martins, Milton Henriques Guimarães-Júnior, Natália da Cunha Severino Sampaio, Neimy Ramos de Oliveira, Patricia Klarmann Ziegelmann, Pedro Guido Soares Andrade, Pedro Ledic Assaf, Petrônio José de Lima Martelli, Polianna Delfino-Pereira, Raphael Castro Martins, Rochele Mosmann Menezes, Saionara Cristina Francisco, Silvia Ferreira Araújo, Talita Fischer Oliveira, Thainara Conceição de Oliveira, Thaís Lorenna Souza Sales, Thiago Junqueira Avelino-Silva, Yuri Carlotto Ramires, Magda Carvalho Pires, Milena Soriano Marcolino

**Affiliations:** 1 Department of Internal Medicine, Medical School, Universidade Federal de Minas Gerais, Av. Professor Alfredo Balena, Belo Horizonte, 190 Brazil; 2 Department of Medicine, Universidade Federal de Lavras, R. Tomas Antonio Gonzaga, 277, Lavras, Brazil; 3 Department of Statistics, Universidade Federal de Minas Gerais, Av. Presidente Antônio Carlos, Belo Horizonte, 6627 Brazil; 4 Botucatu Medical School, Universidade Estadual Paulista “Júlio de Mesquita Filho”, Av. Prof. Mário Rubens Guimarães Montenegro, s/n, Botucatu, Brazil; 5 Institute for Health Technology Assessment (IATS/ CNPq), R. Ramiro Barcelos, Porto Alegre, 2359 Brazil; 6 Hospital Universitário da Universidade Federal de Santa Maria, Av. Roraima, 1000 Santa Maria, Brazil; 7 Hospital São João de Deus, R. do Cobre, 800 Divinópolis, Brazil; 8 Hospital Regional Antônio Dias, R. Maj. Gote, 1231 Patos de Minas, Brazil; 9 Hospital Nossa Senhora da Conceição and Hospital Cristo Redentor, Av. Francisco Trein, 326 Porto Alegre, Brazil; 10 Hospital Risoleta Tolentino Neves, R. das Gabirobas, 01 Belo Horizonte, Brazil; 11 Hospital Júlia Kubitschek, R. Dr. Cristiano Rezende, 2745 Belo Horizonte, Brazil; 12 Hospital Santo Antônio, Praça Dr. Márcio Carvalho Lopes Filho, 501 Curvelo, Brazil; 13 Hospital Santa Rosália, R. do Cruzeiro, 01 Teófilo Otoni, Brazil; 14 Mucuri Medical School, Universidade Federal dos Vales do Jequitinhonha e Mucuri, R. Cruzeiro, 01 Teófilo Otoni, Brazil; 15 Hospital João XXIII, Av. Professor Alfredo Balena, 400 Belo Horizonte, Brazil; 16 Department of Pediatrics, Medical School, Universidade Federal de Minas Gerais, Av. Professor Alfredo Balena, 190 Belo Horizonte, Brazil; 17 Hospital UNIMED BH, Av. do Contorno, 3097 Belo Horizonte, Brazil; 18 Hospital Mãe de Deus, R. José de Alencar, 286 Porto Alegre, Brazil; 19 Hospital Universitário Canoas, Av. Farroupilha, 8001 Canoas, Brazil; 20 Hospital SOS Cárdio, Rodovia, SC-401, 121 Florianópolis, Brazil; 21 Hospital das Clínicas da Universidade Federal de Pernambuco, Av. Prof. Moraes Rego, 1235 Recife, Brazil; 22 Medical School and University Hospital, Universidade Federal de Minas Gerais, Avenida Professor Alfredo Balena, Belo Horizonte, 190 Brazil; 23 Hospital Universitário Ciências Médicas, R. dos Aimorés, 2896 Belo Horizonte, Brazil; 24 Hospital São Lucas da PUCRS, Av. Ipiranga, 6690 Porto Alegre, Brazil; 25 Hospital Luxemburgo, R. Gentios, 1350 Belo Horizonte, Brazil; 26 Hospital Metropolitano Odilon Behrens, R. Formiga, 50 Belo Horizonte, Brazil; 27 Medical School, Universidade Federal de Ouro Preto, R. Diogo de Vasconcelos, 122 Ouro Preto, Brazil; 28 Hospital Bruno Born, Av. Benjamin Constant, 881 Lajeado, Brazil; 29 Hospital Moinhos de Vento, R. Ramiro Barcelos, 910 Porto Alegre, Brazil; 30 Hospital Santa Cruz, R. Fernando Abott, 174 Santa Cruz do Sul, Brazil; 31 Laboratorio de Investigacao Medica em Envelhecimento (LIM-66), Serviço de Geriatria, Hospital das Clínicas HCFMUSP, Faculdade de Medicina, Universidade de Sao Paulo, Sao Paulo, Brazil; 32 Research Institute, Hospital Sirio-Libanes, Sao Paulo, Brazil; 33 Hospitais da Rede Mater Dei, Av. do Contorno, 9000 Belo Horizonte, Brazil; 34 Hospital Márcio Cunha, Av. Kiyoshi Tsunawaki, 48 Ipatinga, Brazil; 35 Hospital Eduardo de Menezes, R. Dr. Cristiano Rezende, 2213 Belo Horizonte, Brazil; 36 Hospital Tacchini, R. Dr. José Mário Mônaco, 358 Bento Gonçalves, Brazil; 37 Hospital Semper, Alameda Ezequiel Dias, 389 Belo Horizonte, Brazil; 38 Hospital Metropolitano Doutor Célio de Castro, R. Dona Luiza, 311 Belo Horizonte, Brazil; 39 Universidade Federal de São João del-Rei, R. Sebastião Gonçalves Coelho, 400 Divinópolis, Brazil; 40 Faculdade Israelita de Ciencias da Saúde Albert Einstein, Hospital Israelita Albert Einstein, Sao Paulo, Brazil; 41 Telehealth Center, University Hospital, Universidade Federal de Minas Gerais, Av. Professor Alfredo Balena, 110 Belo Horizonte, Brazil

**Keywords:** Acute kidney injury, COVID-19, Kidney replacement therapy, Score, Risk factors, Risk prediction

## Abstract

**Background:**

Acute kidney injury (AKI) is frequently associated with COVID-19, and the need for kidney replacement therapy (KRT) is considered an indicator of disease severity. This study aimed to develop a prognostic score for predicting the need for KRT in hospitalised COVID-19 patients, and to assess the incidence of AKI and KRT requirement.

**Methods:**

This study is part of a multicentre cohort, the Brazilian COVID-19 Registry. A total of 5212 adult COVID-19 patients were included between March/2020 and September/2020. Variable selection was performed using generalised additive models (GAM), and least absolute shrinkage and selection operator (LASSO) regression was used for score derivation. Accuracy was assessed using the area under the receiver operating characteristic curve (AUC-ROC).

**Results:**

The median age of the model-derivation cohort was 59 (IQR 47–70) years, 54.5% were men, 34.3% required ICU admission, 20.9% evolved with AKI, 9.3% required KRT, and 15.1% died during hospitalisation. The temporal validation cohort had similar age, sex, ICU admission, AKI, required KRT distribution and in-hospital mortality. The geographic validation cohort had similar age and sex; however, this cohort had higher rates of ICU admission, AKI, need for KRT and in-hospital mortality. Four predictors of the need for KRT were identified using GAM: need for mechanical ventilation, male sex, higher creatinine at hospital presentation and diabetes. The MMCD score had excellent discrimination in derivation (AUROC 0.929, 95% CI 0.918–0.939) and validation (temporal AUROC 0.927, 95% CI 0.911–0.941; geographic AUROC 0.819, 95% CI 0.792–0.845) cohorts and good overall performance (Brier score: 0.057, 0.056 and 0.122, respectively). The score is implemented in a freely available online risk calculator (https://www.mmcdscore.com/).

**Conclusions:**

The use of the MMCD score to predict the need for KRT may assist healthcare workers in identifying hospitalised COVID-19 patients who may require more intensive monitoring, and can be useful for resource allocation.

**Supplementary Information:**

The online version contains supplementary material available at 10.1186/s12916-022-02503-0.

## Background

Coronavirus disease 19 (COVID-19) is mild in most cases, but it can be severe and critical, with multiple organ dysfunction, septic shock and death [1]. Kidney disease among patients with COVID-19 can manifest as acute kidney injury (AKI), hematuria or proteinuria, and it has been associated with an increased risk of mortality [2].

The incidence of AKI among hospitalised patients with COVID-19 has shown to be variable, depending upon the severity of the disease and whether they are outpatient, in the ward or intensive care unit (ICU) environment. A recent systematic review, which included 30 studies and 18,043 patients with COVID-19, observed an overall incidence of AKI of 9.2% (95% confidence interval [CI] 4.6–13.9%), and 32.6% (95% CI 8.5–56.6%) in the ICU [3]. Another systematic review from the beginning of the pandemic included 79 studies and 49,692 patients, and observed a significant variation in the incidence of AKI and kidney replacement therapy (KRT) and the risk of death in patients who develop AKI depending on the continent. The incidence of AKI, KRT requirement and death in patients with AKI was 4.3, 1.4 and 33.3% in Asia, 11.6, 5.7 and 29.4% in Europe and 22.6, 4.0 and 7.4% in North America, respectively [4]. There is a lack of studies from large cohorts in Latin America, which was severely hit by the pandemic.

Previous studies have explored the factors associated with AKI development in COVID-19 patients, including advanced age; black race; underlying medical conditions such as diabetes mellitus, cardiovascular disease, chronic kidney disease and hypertension; COVID-19 severity; use of vasopressor medications and mechanical ventilation requirement [4, 5]. However, most studies are limited to univariate analysis or have small sample sizes and there is a lack of studies analysing independent risk factors for KRT requirement.

A risk score to predict KRT requirement during hospitalisation, using clinical and laboratory data upon hospital presentation may be very useful aiming at a better allocation of health resources. However, there is a lack of evidence in this context. Fang et al. used a score created before the pandemic (UCSD-Mayo risk score) and analysed its efficiency in predicting hospital-acquired AKI in patients with COVID-19, but the performance of the score in patients in ICUs or under mechanical ventilation was not satisfactory [6].

Therefore, we aimed to assess the incidence of AKI and KRT requirement in COVID-19 in-hospital patients, as well as to develop and validate a score to predict the risk of the need for KRT.

## Methods

### Source of data and participants

This cohort study is a substudy of the Brazilian COVID-19 Registry, which included consecutive patients ≥18 years old, hospitalised with COVID-19 confirmed by laboratory test according to WHO criteria, admitted from March to September 2020 in 37 Brazilian hospitals, located in 17 cities, from five Brazilian states. Additionally, patients from the COVID-19 and Frailty (CO-FRAIL) Study were included as the external (geographic) validation cohort [7]. This cohort includes patients > 50 years old, admitted to Sao Paulo University Hospital from March 30 to July 7, 2020.

**Fig. 1:**
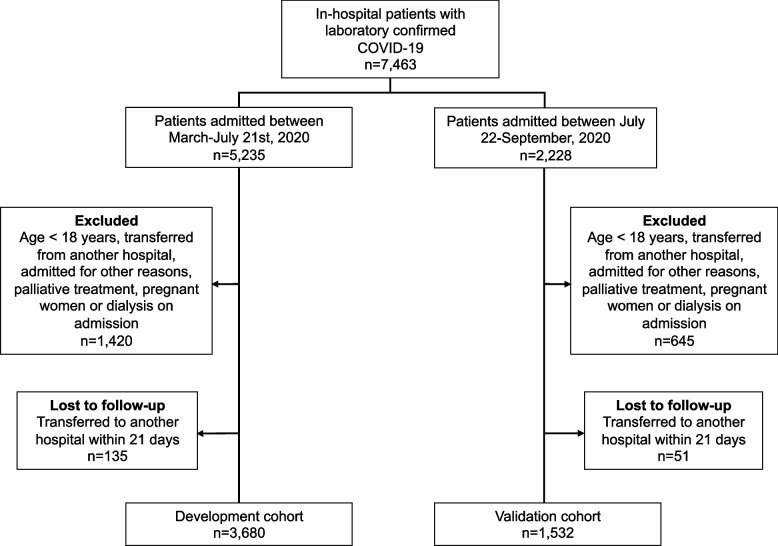
Flowchart of COVID-19 patients included in the study

For the present analysis, patients with chronic kidney disease stage 5 in dialysis previous to COVID-19, pregnant women, undergoing palliative care, admitted with another diagnosis and developed COVID-19 after admission and/or those who were transferred to other hospitals (not part of the multicenter study) during hospitalisation were not included. Two hospitals that did not comply with the study protocol were excluded (Fig. 1).

Model development, validation and reporting followed guidance from the Transparent Reporting of a Multivariable Prediction Model for Individual Prediction or Diagnosis (TRIPOD) checklist (Additional file 1: Table S1) and Prediction model Risk Of Bias Assessment Tool (PROBAST) [8, 9].

### Data collection

Data were extracted from the medical records in participant hospitals, including patient demographic information, comorbidities, laboratory results, treatments (including KRT) and outcomes, as it was previously published in the study protocol [10]. Data were collected by using a prespecified case report form applying Research Electronic Data Capture (REDCap) tools. Variables used in the risk score were obtained at hospital presentation, with the exception of the need for invasive mechanical ventilation, which may have occurred at any time during the hospital stay, except in those patients in which it was initiated after KRT requirement. Indications for invasive mechanical ventilation were defined according to the recommendations of the Brazilian Guidelines [11].

### Clinical outcome

The primary endpoint was KRT requirement. Secondary endpoints were the incidence of AKI and mortality in patients who required KRT.

AKI was defined by an increase in serum creatinine level ≥ 0.3 mg/dl within 48 h or by 50% within 7 days [12]. Indications for acute KRT included clinical manifestations of uremia (such as pericarditis, encephalopathy or an otherwise unexplained decline in mental status); refractory laboratorial abnormalities composed of azotemia (blood urea nitrogen [BUN] >100 mg/dL), a serum potassium level of 6.0 mmol or more per litre, a pH of 7.20 or less and a serum bicarbonate level of 12 mmol per litre or less; or evidence of severe respiratory failure based on a ratio of the partial pressure of arterial oxygen to the fraction of inspired oxygen of 150 or less and clinical perception of volume overload [13]. The indication for the need of KRT was defined by the nephrologist of each participating hospital, as well as the prescription of dialysis treatment.

### Statistical analysis

In the descriptive analyses, categorical variables were described as absolute and relative frequency, and continuous variables by median and quartiles.

The dataset was split into development and validation, according to the date of hospital admission, using July 21, 2020, as the temporal cut (temporal validation).

All analyses were performed using R software version 4.0.2, with the mgcv, finalfit, mice, glmnet, pROC, rms, rmda and psfmi packages. A *p*-value<0.05 was considered statistically significant for all analyses and 95% confidence intervals were reported.

### Missing data

Predictors were imputed if they had up to two thirds of complete values. Variables with a higher proportion of missing values than that were not included in the analysis. After analysing missing data patterns, multiple imputation with chained equations (MICE) was used to handle missing values on candidate variables, considering missing at random. Outcomes were not imputed. Predictive mean matching (PMM) method was used for imputation of continuous predictors and polytomous regression for categorical variables. The results of ten imputed datasets, each with ten iterations, were then combined, following Rubin’s rules [14].

### Development of the risk score model

Predictor selection was based on clinical reasoning and literature review before modelling, as recommended [8]. The development cohort included patients admitted before July 21, 2020.

Variable selection was performed using generalised additive models (GAM), evaluating the relationships between KRT requirement and continuous (through penalised thin plate splines) and categorical (as linear components) predictors and calculating D1- (multivariate Wald test) and D2-statistic (pools test statistics from the repeated analyses).

As our aim was to develop a score for easy application at bedside, continuous variables were categorised on cut-off points, based on evidence from an established score for sepsis [9, 15].

Subsequently, least absolute shrinkage and selection operator (LASSO) logistic regression was used to derive the score by scaling the (L1 penalised) shrunk coefficients (Additional file 2: Table S2). Ten-fold cross-validation methods based on mean squared error criterion were used to choose the penalty parameter *λ* in LASSO.

Lastly, risk groups were proposed based on predicted probabilities: non-high (up to 14.9%), high (15.0–49.9%) and very high risk (≥50.0%).

The specific risks can be easily assessed using the developed MMCD risk score web-based calculator (https://www.mmcdscore.com), which is freely available to the public, and it can also be assessed through infographics (Additional file 3: Figure S1).

### Model validation

External validation comprehended temporal and geographic validation. Patients who were admitted in participant hospitals from July 22, 2020, to September 2020 were included as the temporal validation cohort.

Independent external (geographic) validation was also performed. The analysis included a cohort of patients from São Paulo University Hospital, admitted from March 30 to July 7, 2020 [7]. Inclusion and exclusion criteria were the same as aforementioned.

### Performance measures

To assess model calibration, predicted dialysis probabilities were plotted against the observed values. To assess model discrimination, the area under the receiver operating characteristic curve (AUROC) was calculated, with the respective confidence interval (95% CI), obtained through 2000 bootstrap samples. Positive and negative predictive values of the derived risk groups were also calculated. The Brier score was used to assess the overall performance [16].

## Results

### Participants

**Fig. 2:**
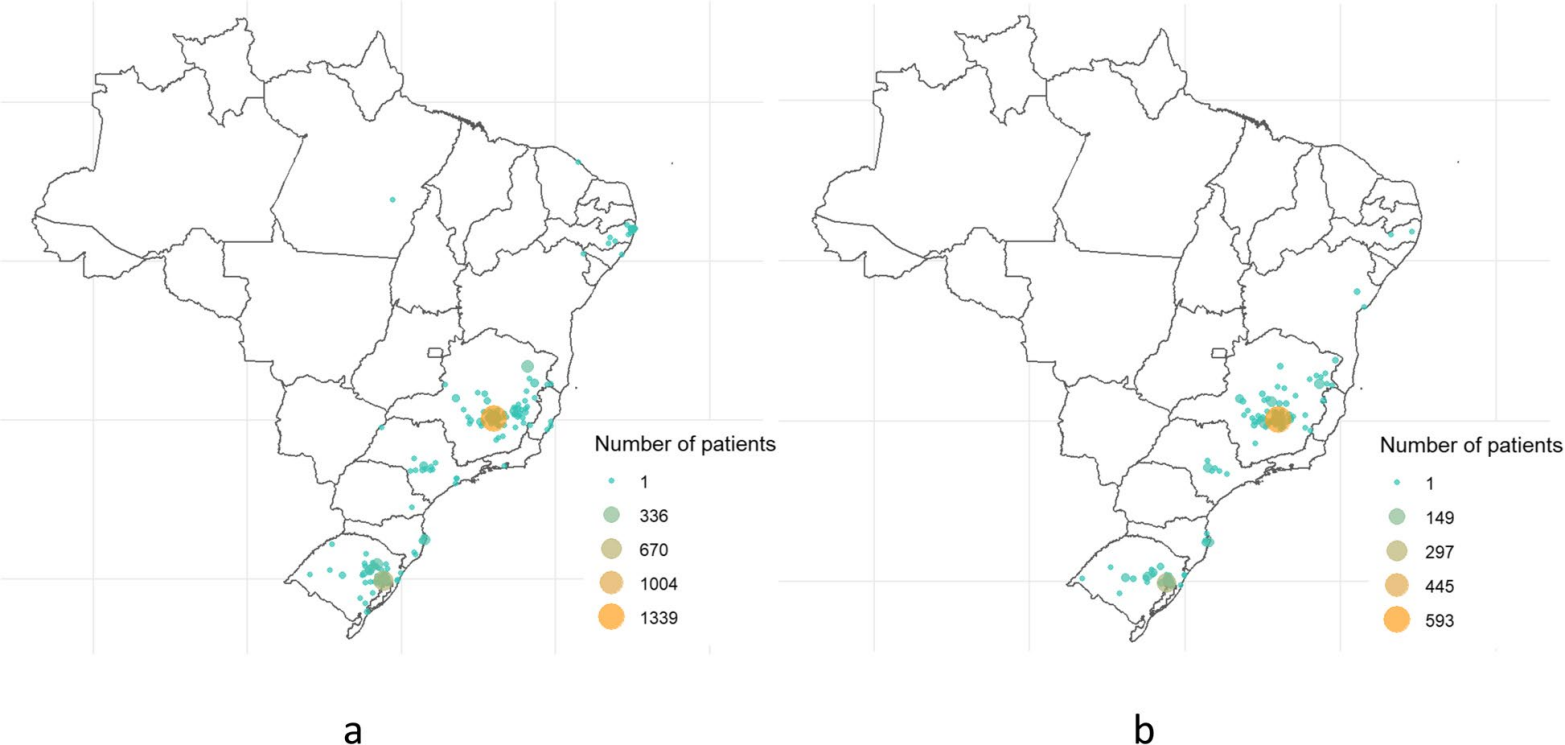
City of residence of patients from **a** development and **b** temporal validation cohorts

The derivation cohort included 3680 COVID-19 patients admitted to the 35 participating hospitals, from March 1, 2020, to July 21, 2020. Those patients were from 159 cities in Brazil (Fig. 2). The median age was 59 (IQR 47–70) years, 54.5% were men, 20.9% evolved with AKI, 9.3% required KRT, and 15.1% died during hospitalisation. Patient demographics, underlying medical conditions, clinical characteristics and laboratory values upon hospital presentation for the derivation and validation cohorts are displayed in Table 1.

**Table 1:** Demographic and clinical characteristics for derivation and validation cohorts of patients admitted to participant hospitals with COVID-19 (*n*=6490)

Label	Derivation cohort		Temporal validation cohort		Geographic validation cohort	
Characteristic	*N* = 3680^a^	Non missing cases (%)	*N* = 1532^a^	Non missing cases (%)	*N* = 1378^a^	Non missing cases (%)
Age (years)	59.0 (47.0, 70.0)	3680 (100%)	62.0 (48.0, 72.0)	1532 (100%)	64.0 (58.0, 72.0)	1378 (100%)
Sex at birth		3680 (100%)		1532 (100%)		1378 (100%)
Men	2,004 (54.5%)		869 (56.7%)		812 (58.9%)	
Comorbidities						
Hypertension	1,977 (53.7%)	3680 (100%)	822 (53.7%)	1532 (100%)	967 (70.2%)	1378 (100%)
Coronary artery disease	180 (4.9%)	3680 (100%)	76 (5.0%)	1532 (100%)	187 (13.6%)	1378 (100%)
Heart failure	224 (6.1%)	3680 (100%)	74 (4.8%)	1532 (100%)	188 (13.6%)	1378 (100%)
Atrial fibrillation/flutter	105 (2.9%)	3680 (100%)	47 (3.1%)	1532 (100%)	74 (5.4%)	1378 (100%)
Stroke	106 (2.9%)	3680 (100%)	53 (3.5%)	1532 (100%)	85 (6.2%)	1378 (100%)
COPD	198 (5.4%)	3680 (100%)	94 (6.1%)	1532 (100%)	100 (7.3%)	1378 (100%)
Diabetes mellitus	1,009 (27.4%)	3680 (100%)	453 (29.6%)	1532 (100%)	616 (44.7%)	1378 (100%)
Obesity (BMI ≥ 30kg/m^2^)	712 (19.3%)	3680 (100%)	273 (17.8%)	1532 (100%)	454 (32.9%)	1378 (100%)
Cirrhosis	17 (0.5%)	3680 (100%)	9 (0.6%)	1532 (100%)	42 (3.0%)	1378 (100%)
Cancer	170 (4.6%)	3680 (100%)	75 (4.9%)	1532 (100%)	147 (10.7%)	1378 (100%)
Number of Comorbidities^b^		3680 (100%)		1532 (100%)		1378 (100%)
0	1,128 (30.7%)		434 (28.3%)		160 (11.6%)	
1	1,107 (30.1%)		501 (32.7%)		336 (24.4%)	
2	923 (25.1%)		392 (25.6%)		394 (28.6%)	
3	384 (10.4%)		149 (9.7%)		298 (21.6%)	
≥ 4	138 (3.8%)		56 (3.7%)		126 (9.1%)	
Clinical assessment at admission						
SF ratio	433.3 (339.3, 452.4)	3582 (97%)	433.3 (342.9, 452.4)	1506 (98%)	202.7 (107.0, 375.0)	1378 (100%)
Heart rate (bpm)	88.0 (78.0, 100.0)	3545 (96%)	87.0 (77.0, 100.0)	1489 (97%)	87.0 (77.0, 98.0)	1378 (100%)
Respiratory rate (irpm)	20.0 (18.0, 24.0)	3043 (83%)	20.0 (18.0, 24.0)	1255 (82%)	24.0 (20.0, 28.0)	1378 (100%)
Glasgow coma scale	15.0 (15.0, 15.0)	3460 (94%)	15.0 (15.0, 15.0)	1441 (94%)	NA	NA
Systolic blood pressure		3524 (96%)		1489 (97%)		1378 (100%)
≥ 90 (mm Hg)	3,338 (94.7%)		1,413 (94.9%)		1,092 (79.2%)	
< 90 (mm Hg)	45 (1.3%)		23 (1.5%)		20 (1.5%)	
Inotrope requirement	141 (4.0%)		53 (3.6%)		266 (19.3%)	
Diastolic blood pressure		3489 (95%)		1481 (97%)		1378 (100%)
> 60 (mm Hg)	2,911 (83.4%)		1,236 (83.5%)		986 (71.6%)	
≤ 60 (mm Hg)	437 (12.5%)		192 (13.0%)		126 (9.1%)	
Inotrope requirement	141 (4.0%)		53 (3.6%)		266 (19.3%)	
Mechanical ventilation at admission	183 (5.0%)	3676 (100%)	63 (4.1%)	1530 (100%)	393 (28.5%)	1378 (100%)
Mechanical ventilation after admission	774 (21.0%)	3680 (100%)	276 (18.0%)	1532 (100%)	266 (19.3%)	1378 (100%)
Laboratory parameters						
Haemoglobin (g/L)	13.4 (12.2, 14.5)	3534 (96%)	13.4 (12.1, 14.6)	1491 (97%)	12.4 (10.9, 13.7)	1354 (98%)
Platelet count (109/L)	193,500.0 (152,000.0, 252,000.0)	3497 (95%)	203,000.0 (156,000.0, 263,850.0)	1478 (96%)	226,000.0 (165,000.0, 304,000.0)	1354 (98%)
Neutrophils-to-lymphocytes ratio	4.4 (2.7, 7.4)	3447 (94%)	4.9 (2.9, 8.2)	1437 (94%)	7.5 (4.2, 13.4)	1352 (98%)
Lactate value	1.4 (1.0, 1.8)	2420 (66%)	1.5 (1.1, 2.0)	999 (65%)	1.6 (1.2, 2.0)	1147 (83%)
C reactive protein (mg/L)	71.0 (34.0, 134.6)	3178 (86%)	73.5 (35.5, 134.8)	1311 (86%)	NA	NA
Blood urea nitrogen (mg/dL)	33.0 (24.0, 47.0)	3310 (90%)	37.0 (27.2, 52.8)	1376 (90%)	48.0 (32.0, 77.0)	1352 (98%)
Creatinine (mg/dL)	0.9 (0.7, 1.2)	3417 (93%)	1.0 (0.8, 1.2)	1434 (94%)	1.0 (0.8, 1.7)	1352 (98%)
Sodium (mmol/L)	137.0 (135.0, 140.0)	3215 (87%)	137.0 (134.9, 140.0)	1356 (89%)	138.0 (135.0, 141.0)	1353 (98%)
Bicarbonate (mEq/L)	23.2 (21.2, 25.2)	2957 (80%)	23.0 (21.0, 25.0)	1217 (79%)	25.0 (22.0, 27.0)	1294 (94%)
pH	7.4 (7.4, 7.5)	2968 (81%)	7.4 (7.4, 7.5)	1217 (79%)	7.4 (7.3, 7.4)	1294 (94%)
Arterial pO_2_	75.0 (63.8, 94.0)	2927 (80%)	74.3 (63.0, 93.7)	1203 (79%)	69.2 (58.5, 84.5)	1092 (79%)
Arterial pCO_2_	35.0 (31.9, 39.0)	2940 (80%)	34.0 (30.9, 38.0)	1205 (79%)	38.3 (33.5, 45.6)	1092 (79%)
Dialysis	343 (9.3%)	3680 (100%)	128 (8.4%)	1532 (100%)	278 (20.2%)	1378 (100%)
In-hospital mortality	554 (15.1%)	3679 (100%)	229 (14.9%)	1532 (100%)	462 (33.5%)	1378 (100%)

^a^Statistics presented: *n* (%); Median (IQR), *COPD* chronic obstructive pulmonary disease, *SF ratio* SpO2/FiO2 ratio, *BMI* body mass index, *NA* not available. ^b^Comorbidities included hypertension, diabetes mellitus, obesity, coronary artery disease, heart failure, atrial fibrillation or flutter, cirrhosis, chronic obstructive pulmonary disease, cancer and previous stroke

Among the patients in the derivation cohort, 1261 (34.3%) required ICU admission. Of those,16.7% developed AKI and 9.1% required KRT, with a mortality rate of 64.7% and 76.7%, respectively.

### Model development

Sixty-three potential risk predictor variables collected at hospital presentation were identified (Additional file 4: Table S3) [17–26]. Of those, 20 were excluded for high collinearity and 11 for high number of missings variables. Consequently, 32 variables were tested.

Four important predictors of the need for KRT during hospitalization were identified using GAM: need for mechanical ventilation, male sex, higher creatinine at hospital presentation and diabetes. Continuous selected predictors were categorised for LASSO logistic regression due to the need for a bedside use score (Table 2). Serum creatinine levels were categorised according to the Sequential Organ Failure Assessment Score (SOFA) [15]. The sum of the prediction scores ranges between 0 and 23, with a high score indicating higher risk of dialysis. Three risk groups were defined based on predicted probabilities of KRT requirement: non-high risk (0–10 score, observed KRT rate 0.4%), high risk (11–14 score, 32.8%) and very high risk (15–23 score, 68.0%), as shown in Table 3. Mortality in each risk strata is also shown in Table 3.

**Table 2:** MMCD score for in-hospital KRT requirement in COVID-19 patients

	Variable	MMCD Score
**M**	**Mechanical ventilation anytime during hospital stay**^**a**^	
	No	0
	Yes	11
**M**	**Sex**	
	Women	0
	Men	1
**C**	**Creatinine (mg/dL) upon hospital presentation**	
	< 1.2	0
	1.2–2.0	1
	2.0–3.5	2
	3.5–5.0	4
	≥ 5.0	10
**D**	**Diabetes mellitus**	
	No	0
	Yes	1

^**a**^ Invasive mechanical ventilation, except in those cases the dialysis preceded mechanical ventilation

**Table 3:** Predicted probability of dialysis; and dialysis and mortality rates for MMCD score risk groups

Risk group	Predicted probability of dialysis	Derivation cohort			Validation (temporal) cohort		
		Patients	Dialysis cases	Deaths	Patients	Dialysis cases	Deaths
Non-high (0–10)	0–14.9%	2486	10 (0.4%)	41 (1.6%)	1,106	8 (0.7%)	21 (1.9%)
High (11–14)	15–49.9%	880	299 (32.8%)	445 (50.6%)	298	108 (34.8%)	176 (59.1%)
Very high (15–23)	> 50%	50	34 (68%)	40 (80%)	30	12 (40%)	23 (76.7%)
Overall		3416	343 (9.3%)	526 (15.4%)	1434	128 (8.4%)	220 (15.3%)

### Model performance

**Fig. 3:**
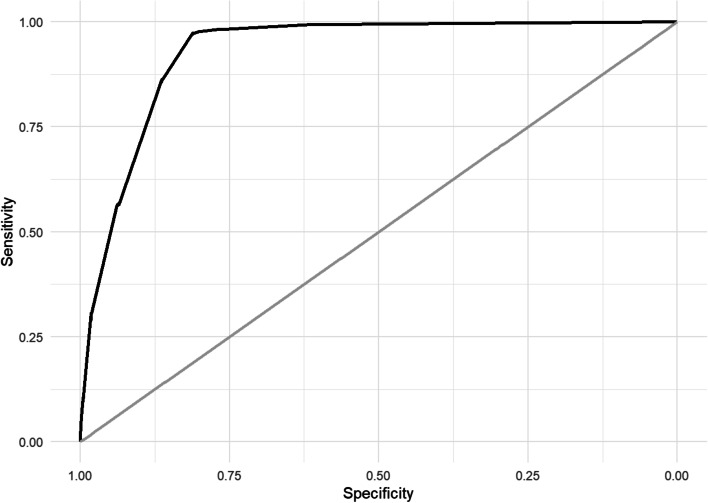
ROC curve from derivation cohort

Discrimination and model overall performance in derivation and validation cohorts for GAM, LASSO and MMCD score are shown in Table 4. Within the derivation cohort, the MMCD risk score showed excellent discrimination (AUROC= 0.929; 95% CI 0.918–0.939) and good overall performance (Brier score: 0.057) (Fig. 3).

**Table 4:** Discrimination and model overall performance in derivation and validation cohorts

Models	Derivation cohort		Temporal validation cohort	
Model	AUROC (95% CI)	Brier score	AUROC (95% CI)	Brier score
GAM	0.938 (0.926–0.947)	0.053	0.917 (0.893–0.937)	0.057
LASSO	0.929 (0.918–0.938)	0.057	0.929 (0.914–0.943)	0.055
MMCD score	0.929 (0.918–0.939)	0.057	0.927 (0.911–0.941)	0.056

### Model validation

A total of 1532 patients admitted between July 22, 2020, and September 31, 2020 were included in the temporal validation cohort. The median age was 62 (IQR 48–72) years; 56.7% were male, 19.8% evolved with AKI, 8.4% required KRT and 14.9% died during hospitalisation. From the total sample, 515 (33.6%) required ICU admission. Of those, 14.6% developed AKI and 8.1% required KRT, with a mortality rate of 65.5% and 82.3%, respectively.

The geographic validation cohort included 1378 patients admitted to São Paulo University Hospital, between March 30 and July 7, 2020. The median age was 64 (IQR 58–72) years; 58.9% were male, 20.2% required KRT, and 33.5% died during hospitalisation (Table 1).

**Fig. 4:**
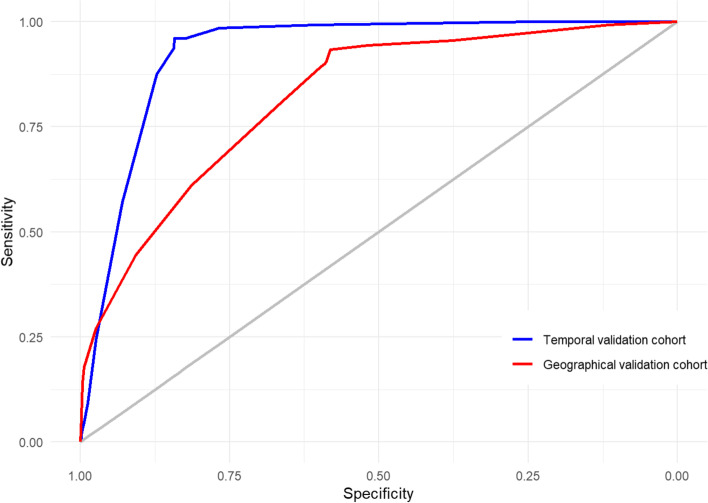
ROC curves from and temporal and geographic validation cohorts

The MMCD Score had a good calibration and performance under temporal and geographic validation cohorts (temporal validation: AUROC 0.927, 95% CI 0.911–0.941, slope = 0.849, Brier score = 0.056 intercept= −0.186; geographic validation: AUROC 0.819, 95% CI 0.792–0.845, slope = 0.560, Brier score = 0.122, intercept = −0.367) (Fig. 4, Additional file 5: Figures S2-S5).

## Discussion

This study included more than 5000 patients hospitalised from a robust cohort of COVID-19 patients from 35 Brazilian hospitals, with external validation in an independent cohort with over 1000 patients. One in every five patients evolved with AKI and 9.3% required KRT. Among the analysed predictors, four variables were related to progression to AKI and KRT requirement, including the need for mechanical ventilation, sex, creatinine upon hospital presentation and diabetes mellitus. The MMCD score had excellent discrimination in derivation and temporal validation cohorts, with AUROC higher than 0.9, a good overall performance.

Renal involvement in COVID-19 infection is complex and probably occurs due to several factors, including direct injury to the renal endothelium, tubular epithelium and podocytes [27]; cytokine storm, with the release of several interleukins and cytokines [3]; cardiorenal syndrome, caused by right ventricular dysfunction secondary to pulmonary infection; hypercoagulable statea; and release of nephrotoxic substances such as creatine phosphokinase secondary to rhabdomyolysis [2].

The need for mechanical ventilation at any time during hospitalisation was an important predictor of progression to AKI and the need for KRT, being the variable with the highest points in the risk score. Scoring mechanical ventilation by itself changed patients’ category to “high risk” for evolving to AKI and KRT requirement. This finding confirms findings from studies carried out in other countries to assess the risk of progression of AKI to KRT in COVID-19 patients, such as USA (OR 10.7 [95% CI 6.81–16.70]) [5] and UK (HR 4.1 [95% CI 1.61–10.49]) [24]. There is a close relationship between alveolar and tubular damage (lung-kidney axis) in acute respiratory distress syndrome (ARDS), often progressing to different degrees of AKI [28]. This is a complex and not fully understood mechanism, probably multifactorial, in which inflammatory mediators are released by ventilated lungs into the systemic circulation [29]. The relationship between mechanical ventilation (MV) and AKI has been widely recognised before the COVID-19 pandemic. Husain-Syed et al. had demonstrated the occurrence of physiological changes triggered by increased intrathoracic pressure secondary to invasive mechanical ventilation that are harmful to the renal function. These changes can cause reduced renal blood flow, glomerular filtration rate and sodium excretion, with a consequent predisposition to progression to AKI and need for KRT [29]. It is difficult to define the specific role that each mechanism plays in the pathogenesis. They are usually observed simultaneously in critically ill patients, which limits the possibility to develop preventive strategies [30].

In studies published by Chan L et al. (*n*=3993) and Fisher M et al. (*n*=3345) with hospitalised patients with COVID-19 in the USA, male sex was considered an independent predictor of progression to AKI and KRT requirement [31, 32], what is in line with our findings. Male sex has been previously observed to be associated with other adverse outcomes in COVID-19 patients.

Creatinine levels upon hospital presentation may be evidence of previous chronic kidney disease or an early manifestation of AKI caused by COVID-19 infection. Chronic kidney disease is a global health problem and a silent disease [33]. Several risk classifications included serum creatinine levels in mortality scores in patients admitted to an intensive care unit (APACHE II, SAPS 3, Sequential Organ Failure Assessment Score [SOFA]), demonstrating the importance of creatinine levels as a marker of severity [15, 34, 35]. In the present analysis, creatinine levels were categorised according to the SOFA score [15] to comply with TRIPOD guidelines, which advises not to use a data-driven method, to avoid model overfitting [9]. Our finding is consistent with a recent systematic review and meta-analysis with 22 studies (*n*=17,391), which observed an increased incidence of AKI in COVID-19 patients hospitalised in the USA who had abnormal baseline serum creatinine levels due to pre-existing chronic kidney disease [36]. Hansrivijit P et al. in their meta-analysis described abnormal basal serum creatinine levels as predictors of progression to AKI [37]. A meta-analysis with 10,335 patients showed that severe cases of COVID-19 had higher serum levels of creatinine and BUN. In severe cases, the risk of progression to need for KRT was 12.99-fold higher compared to non-severe cases, and among patients who died, there was a higher prevalence of AKI, high levels of creatinine and need for KRT [38].

The association between diabetes mellitus and renal dysfunction is well known, in the form of diabetic nephropathy and non inflammatory glomerular damage [39, 40]. In the present analysis, diabetes proved to be a predictor of risk of progression to AKI and KRT requirement in patients hospitalised with COVID-19, which was in line with a recent meta-analysis (26 studies, *n*=5497) [37].

In Brazil, a country severely hit by the pandemic, there is lack of evidence on the association among AKI, need for KRT, mortality and COVID-19. The scarce existing studies are based in small databases. A study published with 200 ICU patients showed a high incidence of AKI (about 50%) and 17% of patients requiring KRT, with significantly higher mortality in patients with AKI and needing KRT, in contrast to patients without AKI and KRT requirement [23]. In our study, the incidence of AKI and need for KRT in ICU patients were lower (about 16 and 9%, respectively), although with higher in-hospital death in this group, similarly to finds in this article. As shown in Table 3, there was a progressive increase in the mortality rate associated with the increase in the score. Patients classified as non-high risk had a mortality of 1.6% in the derivation cohort and 1.9% in the validation cohort, while patients classified as very high risk had a mortality of 80.0% in the derivation cohort and 76.7% in the temporal validation cohort.

The MMCD model retrieved an AUROC of 0.96, which was classified as an excellent discrimination. An American study (*n*=2256) developed prediction models for mechanical ventilation, KRT and readmission in COVID-19 patients using machine learning techniques. Logistic L1 had the best accuracy, although the discrimination results were inferior than the one observed in the present analysis (0.847 [95% CI, 0.772-0.936]). Additionally, the model uses too many risk predictor variables, hindering its applicability in clinical practice [25].

External validation was performed with a cohort of patients referred to a tertiary hospital, most of which were critically ill, with a high rate of ICU admission, use of mechanical ventilation and need for KRT and mortality. As the accuracy of a prediction model is always high, whether the model is validated on the development cohort used to derive the model only, the assessment of accuracy in those studies may be overoptimistic [9].

The criteria for orotracheal intubation evolved over time. Still, we believe it has not affected our findings. The first wave of COVID-19 pandemic in Brazil was in June 2020, late in relation to Europe, which was affected in March 2020. Therefore, when the country faced its first wave, the knowledge about intubation criteria and outcomes had already evolved. The fact that the score’s high accuracy was not reduced in the temporal validation cohort (cut-off on July 21, 2020) is evidence of no significant influence on the results obtained in the temporal validation sample (AUROC 0.927 CI 95% 0.911–0.941).

### Strengths and limitations

Our study used a large patients database to develop a risk score to predict the need for KRT in patients admitted with COVID-19. A major strength of the MMCD score is its simplicity; the use of objective parameters, which may reduce the variability; and easy availability, even in under-resourced settings. Then, the MMCD score may help clinicians to make a prompt and reasonable decision to optimise the management of COVID-19 patients with AKI and potentially reduce mortality. Additionally, its development and validation strictly followed the TRIPOD recommendations [9].

This study has limitations. Indication and timing of initiation of the KRT may differ according to institutional protocols; however, there is a consensus on the criteria on which KRT should be initiated [13]. We did not collect information on patients who did not perform dialysis due to limited resources. Still, this has not affected the accuracy of the score. Additionally, as any other score, MMCD may not be directly generalised to populations from other countries without further validation.

With regard to AKI assessment, it was not possible to use the criterion based on diuresis due to unavailability of this data, as well as the baseline creatinine value to identify AKI due to the lack of data on previous serum creatinine of patients admitted to participating hospitals. Instead, we used the increase of >0.3 mg/dl in creatinine values over 48 h or 1.5-fold increase within 7 days during the hospitalisation, when compared to creatinine at hospital presentation. Therefore, the real incidence of AKI may be underestimated.

Finally, external validation of the MMCD score in other countries should be performed with more recent data on COVID-19 infection, considering the multiple temporal aspects of the pandemic and changes in disease management.

### Possible applications

Using predictors available at baseline and within the first hours of the admission, we could objectively predict the probability of KRT of a COVID-19 patient with AKI. With an accurate prediction, it may help to organise resource allocation to patients who are at the highest risk of KRT requirement [25], in addition to selecting patients who may benefit from renal protection strategies, close assessment and follow-up by a nephrologist [41].

## Conclusions

In conclusion, we developed and validated a clinical prediction score named MMCD, to predict the need for KRT in COVID-19 patients. This score used a few predictors available at baseline and mechanical ventilation anytime during hospital admission, and retrieved a good accuracy. This could be an inexpensive tool to predict the need for KRT objectively and accurately. Additionally, it may be used to inform clinical decisions and the assignment to the appropriate level of care and treatment for COVID-19 patients with AKI.

## Supplementary Information

**Additional file 1: Table S1.** TRIPOD checklist for transparent reporting on a multivariable prognostic model.

**Additional file 2: Table S2.** L1 penalised shrunk coefficients for the MMCD score.

**Additional file 3: Figure S1.** MMCD score risk for adult patients admitted to hospital with COVID-19 – MMCD score infographics.

**Additional file 4: Table S3.** Assessment of potential predictors for the model development.

**Additional file 5: Figure S2.** Calibration slope for the MMCD score. **Figure S3.** Combined decision curve for the MMCD score. **Figure S4.** Calibration slope for the MMCD score in geographic validation. **Figure S5.** Combined decision curve for the MMCD score in geographic validation.

## Data Availability

Any additional data pertaining to this manuscript are available from the corresponding author upon reasonable request.

## Abbreviations

AKI: Acute kidney injury
ARDS: Acute respiratory distress syndrome
AUROC: Area under the receiver operating characteristic
BMI: Body mass index
BPM1: Beats per minute
BPM2: Breaths per minute
BUN: Blood urea nitrogen
CI: Confidence interval
COPD: Chronic obstructive pulmonary disease
COVID-19: Coronavirus disease 19
GAM: Generalised additive models
GBT: Gradient boosted trees
ICU: Intensive care unit
IL-6: Interleukin-6
IQR: Interquartile range
KDIGO: Kidney Disease: Improving Global Outcomes
KRT: Kidney replacement therapy
LASSO: Lleast absolute shrinkage and selection operator
MICE: Multiple imputation with chained equations
MMCD: Mechanical ventilation, male, creatinine, diabetes
MV: Mechanical ventilation
pCO_2_: Partial pressure of carbon dioxide
PEEP: Positive end-expiratory pressure
PMM: Predictive mean matching
PO_2_: Partial pressure of oxygen
PROBAST: Prediction model Risk Of Bias Assessment Tool
REDCap: Research Electronic Data Capture
SBP: Systolic blood pressure
SF ratio: SpO_2_/FiO_2_ ratio
SOFA: Sepsis-related Organ Failure Assessment
TRIPOD: Transparent Reporting of a Multivariable Prediction Model for Individual Prediction or Diagnosis
USA: United States of America
