# Supplementary material for: Development and validation of the MMCD score to predict kidney replacement therapy in COVID-19 patients
Source: BMC Med. 2022 Sep 2;20:324. doi: 10.1186/s12916-022-02503-0 (PMC9438299; doi:10.1186/s12916-022-02503-0)
Supplement: Supplementary file 2 — Additional file 2: Table S2. L1 penalised shrunk coefficients for the MMCD score. [file 12916_2022_2503_MOESM2_ESM.docx]

**Additional file 2.** Development of the risk score model

**Table S2.** L1 penalized shrunk coefficients for the MMCD score

|  | **Variable** | **Coefficient** |
| --- | --- | --- |
| **M** | **Intercept**  **Mechanical ventilation anytime during hospital stay^a^** | -4,841 |
|  | No | - |
|  | Yes | 3,682 |
| **M** | **Sex** |  |
|  | Women | - |
|  | Men | 0,213 |
| **C** | **Creatinine (mg/dL) upon hospital presentation** |  |
|  | < 1.2 | - |
|  | 1.2 - 2.0 | 0,306 |
|  | 2.0 - 3.5 | 0,755 |
|  | 3.5 - 5.0 | 1,444 |
|  | ≥ 5.0 | 3,179 |
| **D** | **Diabetes mellitus** |  |
|  | No | - |
|  | Yes | 0,263 |

**^a^** Except in those cases the dialysis preceded mechanical ventilation.
