# Supplementary material for: Development and validation of the MMCD score to predict kidney replacement therapy in COVID-19 patients
Source: BMC Med. 2022 Sep 2;20:324. doi: 10.1186/s12916-022-02503-0 (PMC9438299; doi:10.1186/s12916-022-02503-0)
Supplement: Supplementary file 3 — Additional file 3: Figure S1. MMCD score risk for adult patients admitted to hospital with COVID-19 – MMCD score infographics. [file 12916_2022_2503_MOESM3_ESM.zip › Additional file 3R3.docx]

**Additional file 3:** MMCD score infographics

**Figure S1:** MMCD score risk for adult patients admitted to hospital with COVID-19
