# Supplementary material for: Development and validation of the MMCD score to predict kidney replacement therapy in COVID-19 patients
Source: BMC Med. 2022 Sep 2;20:324. doi: 10.1186/s12916-022-02503-0 (PMC9438299; doi:10.1186/s12916-022-02503-0)
Supplement: Supplementary file 3 — Additional file 3: Figure S1. MMCD score risk for adult patients admitted to hospital with COVID-19 – MMCD score infographics. [file 12916_2022_2503_MOESM3_ESM.zip › Figure S1R3.pdf]

Figure S1. MMCD score risk for adult patients admitted to hospital with COVID-19

	Points
**Mechanical ventilation anytime during hospital stay[a]**	
No	0
Yes	11
**Sex**	
Women	0
Men	1
**Creatinine (mg/dL) upon hospital presentation**	
< 1.2	0
1.2 - 2.0	1
2.0 - 3.5	2
3.5 - 5.0	4
≥ 5.0	10
**Diabetes mellitus**	
No	0
Yes	1

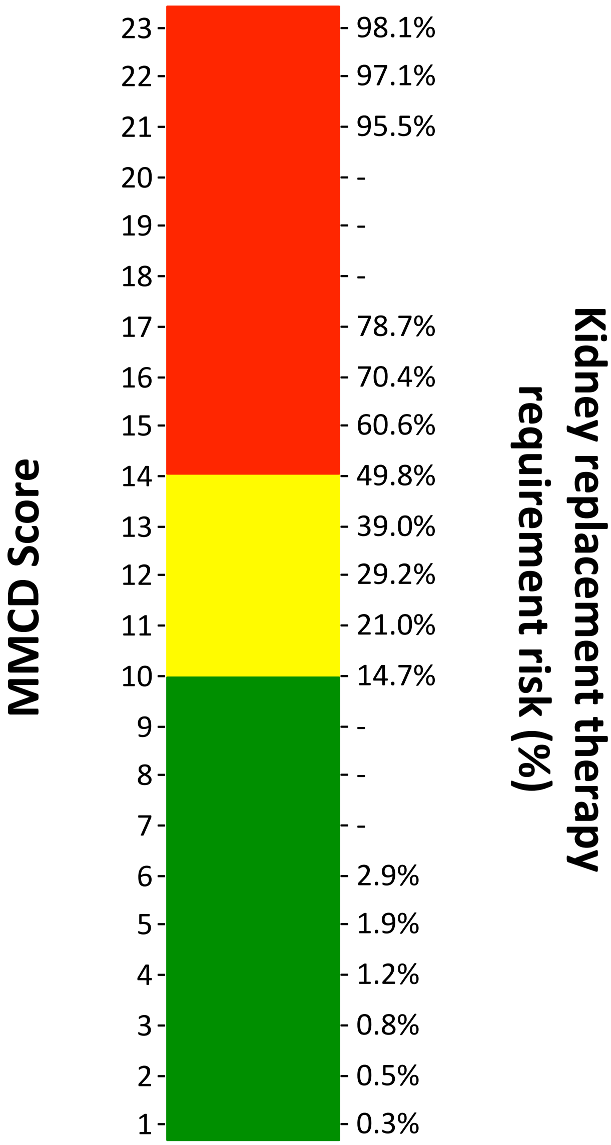

[a] Except in those cases the dialysis preceded mechanical ventilation
