# Supplementary material for: Development and validation of the MMCD score to predict kidney replacement therapy in COVID-19 patients
Source: BMC Med. 2022 Sep 2;20:324. doi: 10.1186/s12916-022-02503-0 (PMC9438299; doi:10.1186/s12916-022-02503-0)
Supplement: Supplementary file 4 — Additional file 4: Table S3. Assessment of potential predictors for the model development. [file 12916_2022_2503_MOESM4_ESM.docx]

**Additional file 4:** Model development: potencial predictors

**Table S3:** Assessment of potential predictors for the model development

| **Variables** | **Scientific evidence** | **Model development (derivation cohort)** |
| --- | --- | --- |
| **Demographic** |  |  |
| Age in years (continuous) | [17] [18] [19] [20] [21] [22] [23] [24] [25] [26] | Included as candidate predictor |
| Sex at birth | [18] [19] [22] [23] [24] [25] [26] | Included as candidate predictor |
| **Conditions highly and moderately associated with increased risk of complications (NHS guidance)** | - | - |
| Cardiovascular system | - | - |
| - Hypertension | [18] [21] [22] [23] [24] [26] | Included as candidate predictor |
| - Coronary artery disease | [18] [19] [21] [22] [23] [24] [26] | Included as candidate predictor |
| - Heart failure | [18] [21] [22] [23] [24] [26] | Included as candidate predictor |
| - Atrial fibrillation/flutter | [19] [21] [24] [26] | Included as candidate predictor |
| - Ischemic stroke | [26] | Included as candidate predictor |
| Diabetes mellitus | [18] [19] [21] [22] [23] [24] [26] | Included as candidate predictor |
| Obesity (BMI>30kg/m2) | [19] [21] [23] [24] [26] | Included as candidate predictor |
| Cirrhosis | [21] [24] [26] | Included as candidate predictor |
| Chronic kidney disease | [18] [19] [24] [25] [26] | Included as candidate predictor |
| HIV infection | [19] [24] [26] | Included as candidate predictor |
| Malignant neoplasm | [18] [19] [21] [23] [24] | Included as candidate predictor |
| Previous transplantation | [26] | Included as candidate predictor |
| Report of hospital surgical procedure in the last 90 days |  | Included as candidate predictor |
| **Lifestyle** |  | Included as candidate predictor |
| Illicit drugs use |  | Included as candidate predictor |
| Alcohol abuse |  | Included as candidate predictor |
| Current smoking | [23] [24] [25] [26] | Included as candidate predictor |
| Previous smoking | [23] [26] | Included as candidate predictor |
| **Clinical findings** | - | - |
| Symptoms time | [23] | Included as candidate predictor |
| Sensory impairment | [26] | High collinearity with Invasive Mechanical Ventilation, not included |
| Glasgow come scale | [26] | High collinearity with Invasive Mechanical Ventilation, not included |
| Mental status | [26] | - |
| - Alert |  | High collinearity with SBP, not included |
| - Confused |  | High collinearity with SBP, not included |
| - Disoriented |  | High collinearity with SBP, not included |
| - Sleepy |  | High collinearity with SBP, not included |
| - Torporous |  | High collinearity with SBP, not included |
| - Coma |  | High collinearity with SBP, not included |
| Systolic blood pressure (mmHg) | [19] [20] [25] [26] | Included as candidate predictor |
| Diastolic blood pressure (mmHg) | [19] [20] [25] [26] | High collinearity with SBP, not included |
| Use of vasoactive amines | [19] [20] [23] [24] [25] [26] | High collinearity with SBP, not included |
| Heart rate (bpm) | [19] [25] [26] | Included as candidate predictor |
| Respiratory rate (bpm) | [19] [25] [26] | Included as candidate predictor |
| O2 saturation (%) | [19] [24] [25] [26] | Included as candidate predictor |
| Invasive Mechanical Ventilation at admission | [19] [22] [23] [24] [25] [26] | Included as candidate predictor |
| Invasive Mechanical Ventilation on third day of hospitalization | [19] [22] [23] [24] [25] | High collinearity with Invasive Mechanical Ventilation, not included |
| Invasive Mechanical Ventilation on fifith day of hospitalization | [19] [22] [23] [24] [25] | High collinearity with Invasive Mechanical Ventilation, not included |
| Invasive Mechanical Ventilation at any time of hospitalization | [19] [22] [23] [24] [25] | High collinearity with Invasive Mechanical Ventilation, not included |
| **Laboratory findings** | - | - |
| Hemoglobin (g/dL) | [19] [23] [24] [25] [26] | High collinearity with NLR, not included |
| Leukocytes (cells/mm3) | [18] [21] [22] [23] [24] [25] | High collinearity with NLR, not included |
| Neutrophils (cels/mm3) | [18] [21] [22] [24] [25] | High collinearity with NLR, not included |
| Lymphocytes (cels/mm3) | [18] [19] [21] [22] [23] [24] [25] | High collinearity with NLR, not included |
| Neutrophil/lymphocyte ratio | [18] [19] [21] [22] | Included as candidate predictor |
| Platelets (cels/mm3) | [18] [19] [22] [23] [24] [25] [26] | High collinearity with NLR, not included |
| Albumin (g/dL) | [19] [21] [22] [25] [26] | Too many missing values, not included |
| Creatinine (mg/dL) | [19] [20] [22] [23] [24] [25] | Included as candidate predictor |
| Creatine phosphokinase (U/L) | [18] [23] [25] | Too many missing values, not included |
| D-dimer (ng/ml) | [22] [23] [25] [26] | Too many missing values, not included |
| Ferritin (ng/mL) | [23] [24] [25] [26] | Too many missing values, not included |
| Lactate dehydrogenase (U/L) | [23] [25] | Too many missing values, not included |
| Protein C reactive (mg/L) | [18] [19] [22] [24] [25] [26] | Included as candidate predictor |
| Procalcitonin (ng/mL) | [22] [23] [25] [26] | Too many missing values, not included |
| Partial thromboplastin time (seconds)/control | [25] | Too many missing values, not included |
| International normalization ratio (INR) | [25] | Too many missing values, not included |
| Sodium (mmoL) | [21] [23] [24] [25] | Included as candidate predictor |
| Aspartate aminotransferase (U/L) | [19] [22] [23] [24] [25] | Too many missing values, not included |
| Alanine aminotransferase (U/L) | [19] [21] [22] [23] [24] [25] | Too many missing values, not included |
| Troponin | [25] [26] | Too many missing values, not included |
| Blood urea nitrogen (mg/dL) | [19] [23] [24] [25] [26] | Included as candidate predictor |
| pH | [23] [24] [25] | Included as candidate predictor |
| arterial pCO2 | [23] [25] | Included as candidate predictor |
| arterial pO2 | [19] [23] [24] [25] [26] | High collinearity with pH, not included |
| Bicarbonate | [19] [23] [24] [25] | High collinearity with pCO2, not included |
