# Supplementary material for: Development and validation of the MMCD score to predict kidney replacement therapy in COVID-19 patients
Source: BMC Med. 2022 Sep 2;20:324. doi: 10.1186/s12916-022-02503-0 (PMC9438299; doi:10.1186/s12916-022-02503-0)
Supplement: Supplementary file 5 — Additional file 5: Figure S2. Calibration slope for the MMCD score. Figure S3. Combined decision curve for the MMCD score. Figure S4. Calibration slope for the MMCD score in geographic validation. Figure S5. Combined decision curve for the MMCD score in geographic validation. [file 12916_2022_2503_MOESM5_ESM.zip › Additional file 5R3.docx]

**Additional file 5:** Calibration and combined decision curves for the MMCD score

**Figure S2:** Calibration slope for the MMCD score

**Figure S3:** Combined decision curve for the MMCD score

**Figure S4:** Calibration slope for the MMCD score in geographic validation

**Figure S5:** Combined decision curve for the MMCD score in geographic validation
