# Supplementary figures and images for: Development and validation of the MMCD score to predict kidney replacement therapy in COVID-19 patients
Source: BMC Med. 2022 Sep 2;20:324. doi: 10.1186/s12916-022-02503-0 (PMC9438299; doi:10.1186/s12916-022-02503-0)

**Figure S2.** Calibration slope for the MMCD score

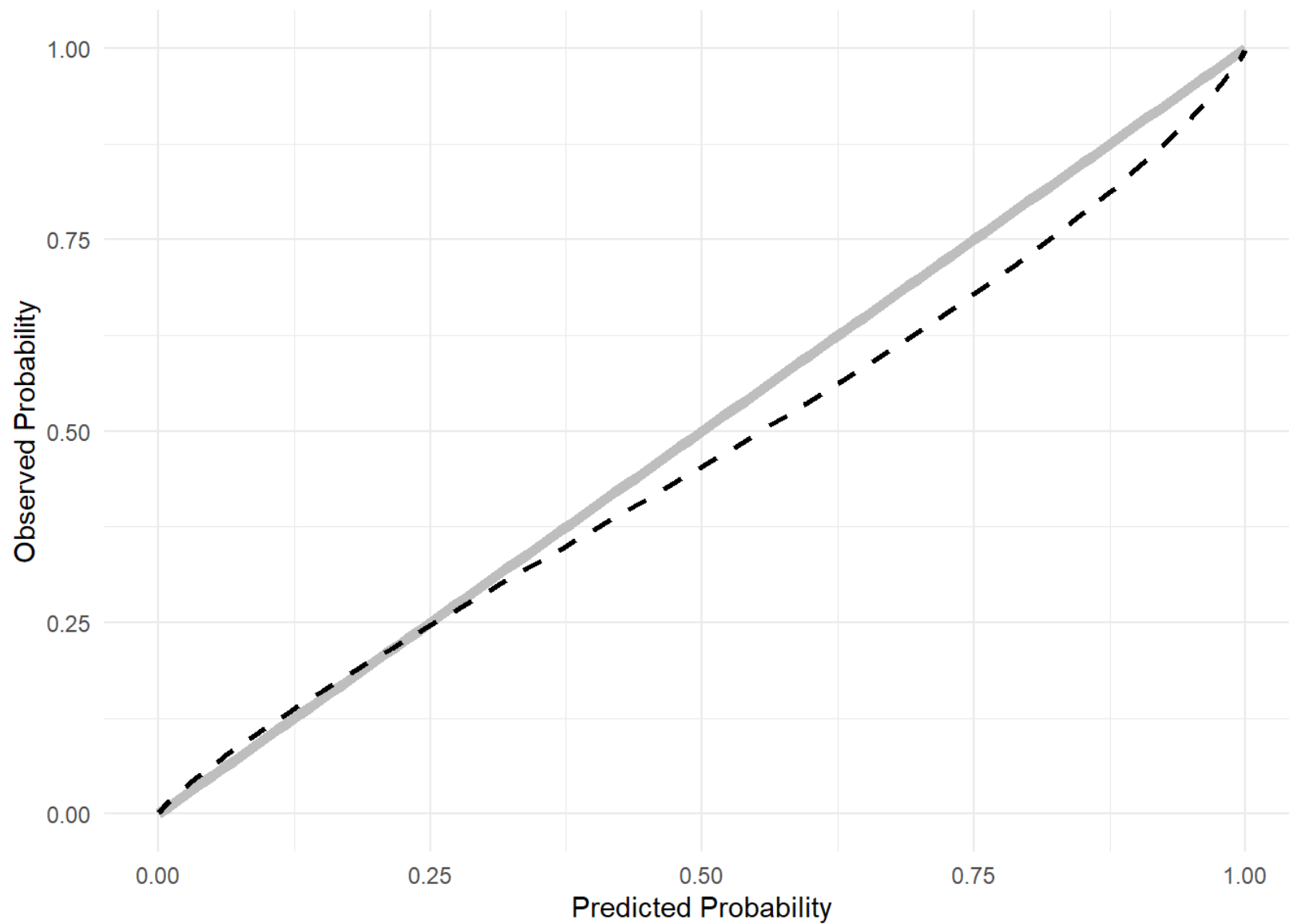

Supplement: Supplementary file 5 — Additional file 5: Figure S2. Calibration slope for the MMCD score. Figure S3. Combined decision curve for the MMCD score. Figure S4. Calibration slope for the MMCD score in geographic validation. Figure S5. Combined decision curve for the MMCD score in geographic validation. [file 12916_2022_2503_MOESM5_ESM.zip › Figure S2R3.pdf]

**Figure S3.** Combined decision curve for the MMCD score

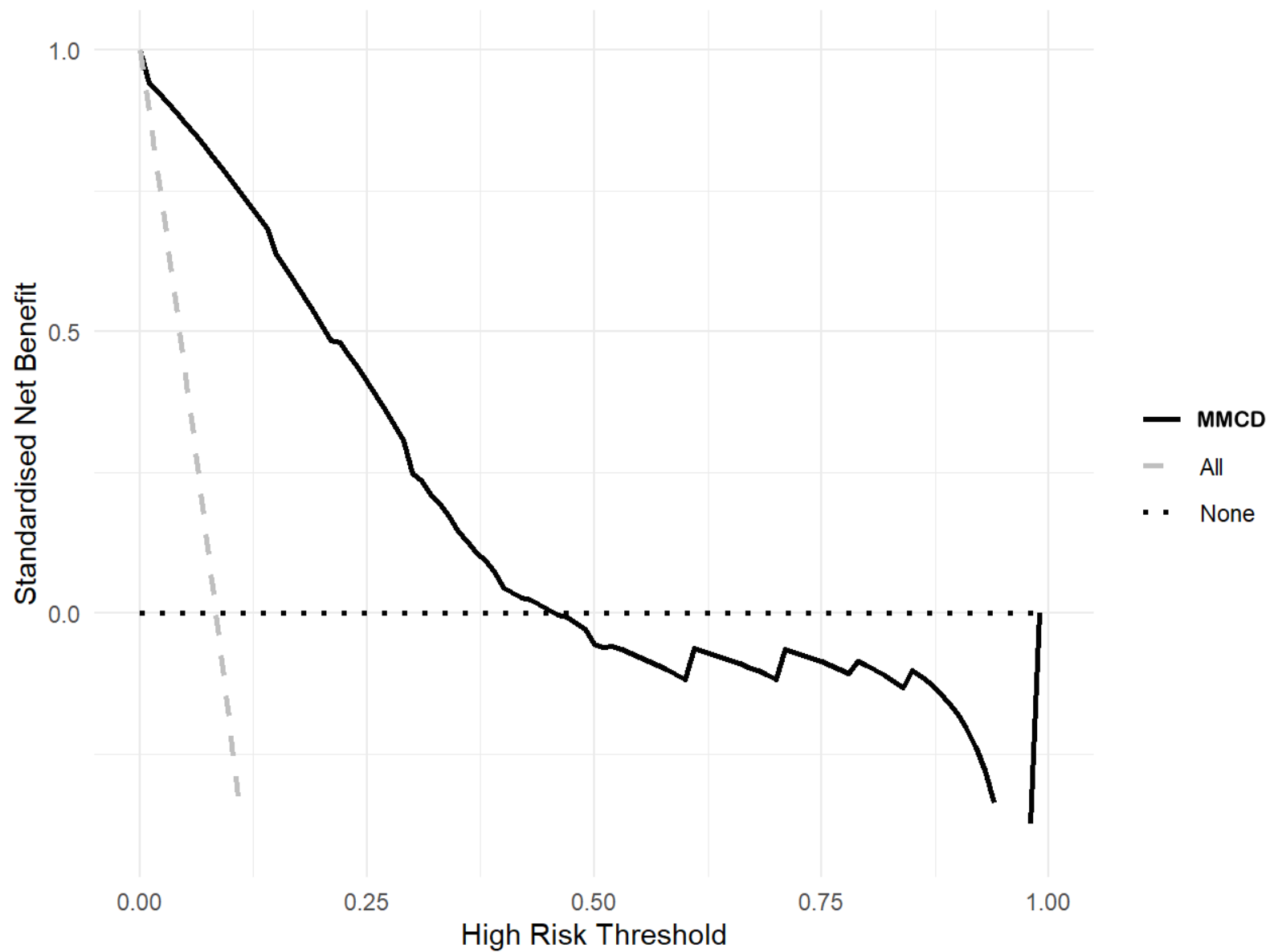

Supplement: Supplementary file 5 — Additional file 5: Figure S2. Calibration slope for the MMCD score. Figure S3. Combined decision curve for the MMCD score. Figure S4. Calibration slope for the MMCD score in geographic validation. Figure S5. Combined decision curve for the MMCD score in geographic validation. [file 12916_2022_2503_MOESM5_ESM.zip › Figure S3R3.pdf]

**Figure S4.** Calibration slope for the MMCD score in geographic validation

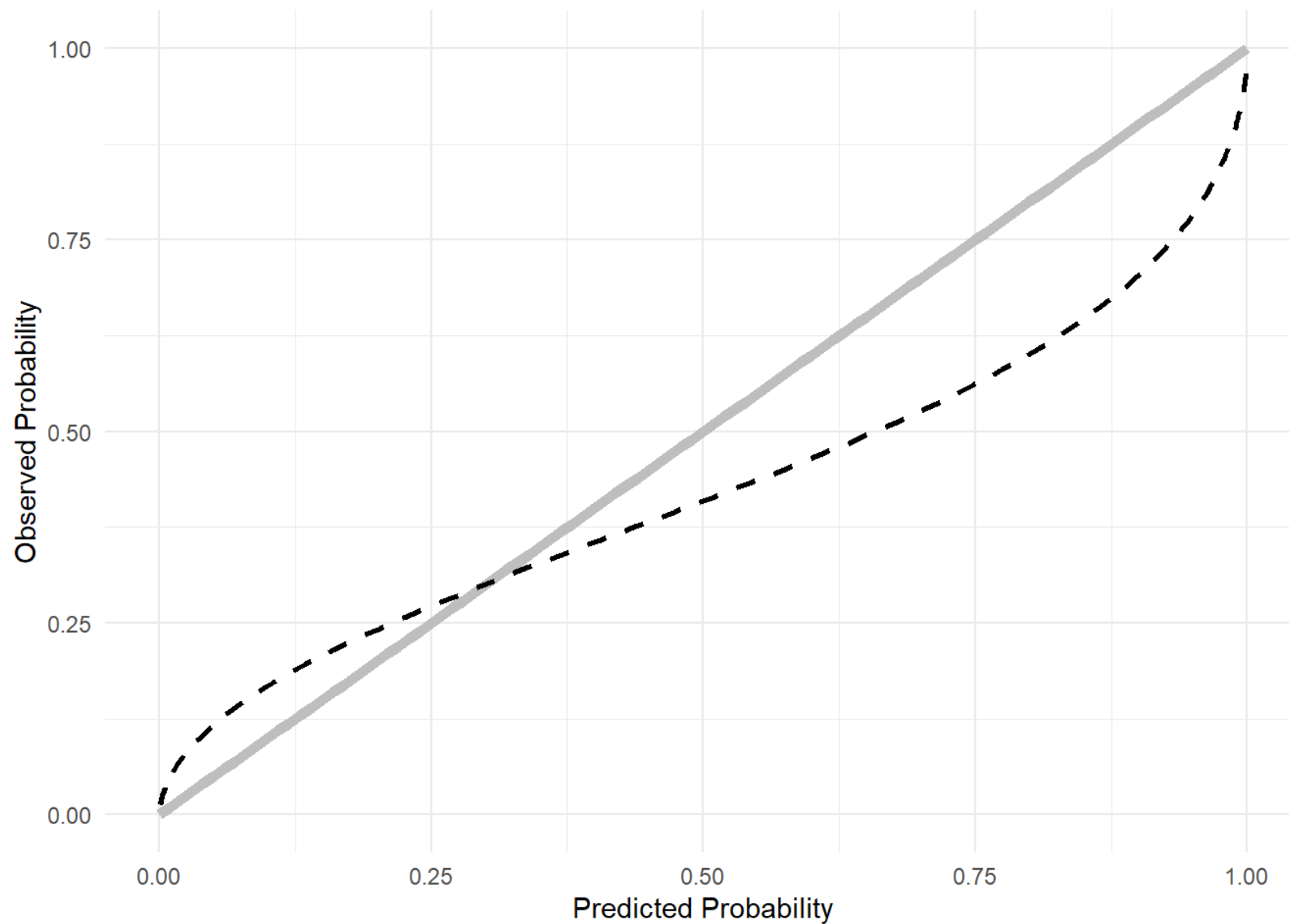

Supplement: Supplementary file 5 — Additional file 5: Figure S2. Calibration slope for the MMCD score. Figure S3. Combined decision curve for the MMCD score. Figure S4. Calibration slope for the MMCD score in geographic validation. Figure S5. Combined decision curve for the MMCD score in geographic validation. [file 12916_2022_2503_MOESM5_ESM.zip › Figure S4R3.pdf]

**Figure S5.** Combined decision curve for the MMCD score in geographic validation

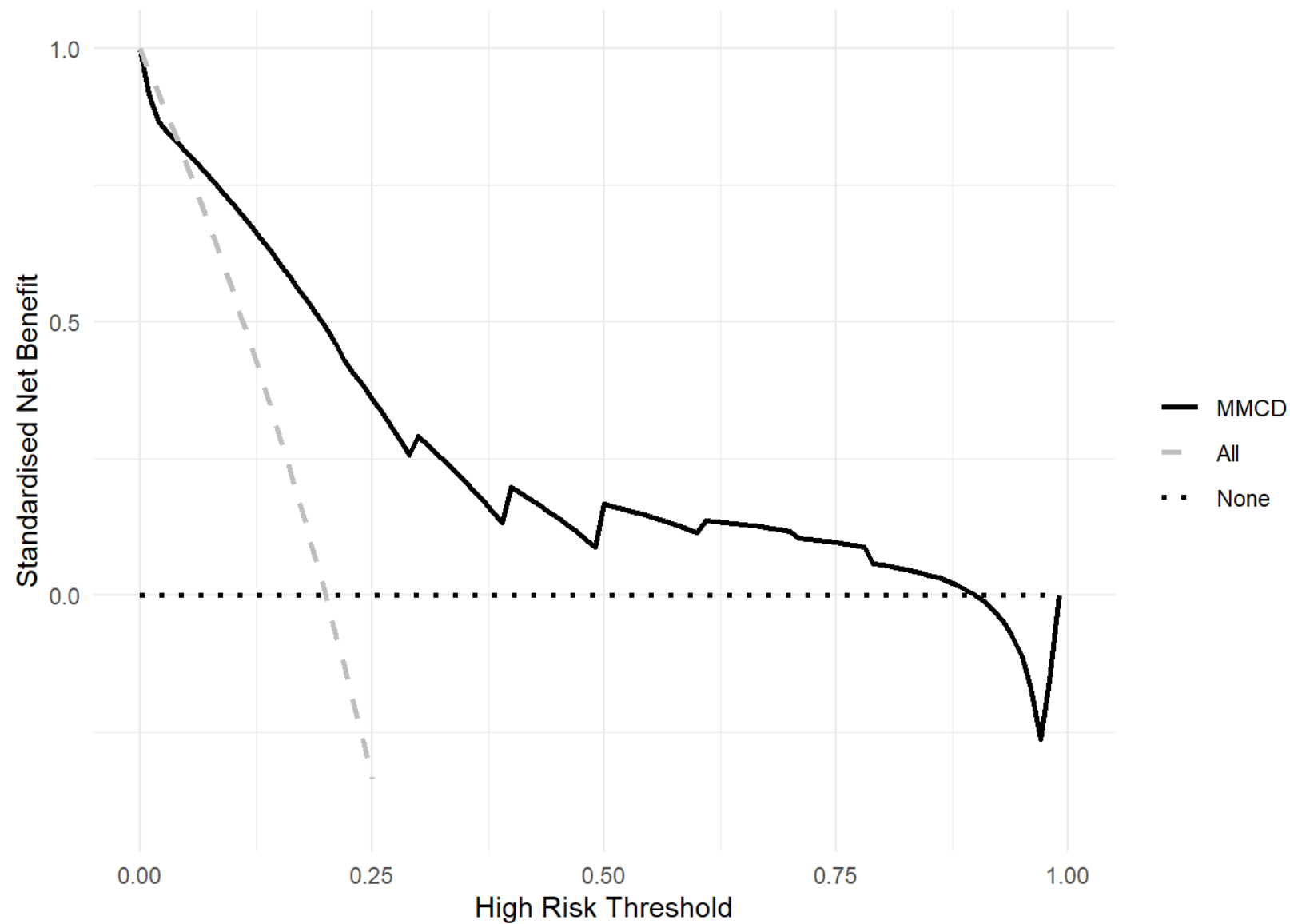

Supplement: Supplementary file 5 — Additional file 5: Figure S2. Calibration slope for the MMCD score. Figure S3. Combined decision curve for the MMCD score. Figure S4. Calibration slope for the MMCD score in geographic validation. Figure S5. Combined decision curve for the MMCD score in geographic validation. [file 12916_2022_2503_MOESM5_ESM.zip › Figure S5R3.pdf]
